# Virtual Reality Technology as an Educational and Intervention Tool for Children with Autism Spectrum Disorder: Current Perspectives and Future Directions

**DOI:** 10.3390/bs12050138

**Published:** 2022-05-10

**Authors:** Minyue Zhang, Hongwei Ding, Meri Naumceska, Yang Zhang

**Affiliations:** 1 Speech-Language-Hearing Center, School of Foreign Languages, Shanghai Jiao Tong University, Shanghai 200240, China; zhang.my@sjtu.edu.cn; 2 70 Flowers Association for Early Intervention and Education of Children with Autism, 1000 Skopje, North Macedonia; merinaumceska@gmail.com; 3 Department of Speech-Language-Hearing Sciences, University of Minnesota, Minneapolis, MN 55455, USA

**Keywords:** virtual reality, autism spectrum disorder, education, intervention, childhood and adolescence

## Abstract

The worldwide rising trend of autism spectrum disorder (ASD) calls for innovative and efficacious techniques for assessment and treatment. Virtual reality (VR) technology gains theoretical support from rehabilitation and pedagogical theories and offers a variety of capabilities in educational and interventional contexts with affordable products. VR is attracting increasing attention in the medical and healthcare industry, as it provides fully interactive three-dimensional simulations of real-world settings and social situations, which are particularly suitable for cognitive and performance training, including social and interaction skills. This review article offers a summary of current perspectives and evidence-based VR applications for children with ASD, with a primary focus on social communication, including social functioning, emotion recognition, and speech and language. Technology- and design-related limitations, as well as disputes over the application of VR to autism research and therapy, are discussed, and future directions of this emerging field are highlighted with regards to application expansion and improvement, technology enhancement, linguistic diversity, and the development of theoretical models and brain-based research.

## 1. Introduction

Autism spectrum disorder (ASD) is a neurodevelopmental condition characterized by difficulties/differences in social communication, interaction, language, cognition, and behavioral activities across a variety of contexts [1]. Although the degree of impairment varies tremendously among individuals with ASD, these symptoms can lead to social exclusion and pose significant obstacles to maintaining and sustaining friendships and employment (in the case of affected adult individuals) [2]. To address the problems, traditional intervention approaches generally require intensive support under the direct supervision of well-trained professionals. However, professional care and amenities are not always accessible to many individuals with ASD, due to unaffordable intervention costs and/or lack of available qualified therapists [3], which calls for the development of new and efficacious tools for ASD assessment and intervention. In recent years, there has been a rapid advance in the development of virtual reality (VR) technology and its uses for leisure and education. VR has also emerged as an effective approach in various areas of the health field, such as diagnosis [4], rehabilitation [5], surgical training [6], and mental health treatment [7]. The wide application of this technology has inspired many researchers to consider the potential and effectiveness of implementing VR technology for the assessment and treatment of ASD [8,9,10,11]. This article attempts to provide an updated review of the emerging field to summarize the current perspectives and identify future directions.

Our review consists of seven sections. Following the brief Introduction, Section 2 summarizes the existing review articles on the use of VR for ASD intervention, points out the differences between them and the present review, and highlights the purpose and the target audience of this article. Section 3 provides the theoretical framework for VR as a powerful technology-driven pedagogical and intervention platform, covering what VR is, the theoretical underpinnings and models for VR training and intervention, its educational and interventional uses for typical and special populations, and popular VR products. Section 4 highlights the core impairments of ASD and its increasing prevalence and discusses the advantages of incorporating VR in ASD research and therapy, as well as the potentials of VR for investigating social interaction. Section 5 illustrates recent evidence-based applications of VR to ASD research and intervention, with specific studies as examples, focusing on social aspects, including social functioning, emotion recognition, and speech and language. Section 6 offers a general discussion, and points out current technology- and design-related limitations, as well as controversial issues of VR, and highlights several promising future directions. Section 7 concludes the article.

## 2. Related Survey Articles

In autism literature, a number of recently published review articles have been devoted to the use of VR for intervention (See Table 1 for a summary). The majority of them adopt the systematic review method for evidence synthesis and focus on a specific well-defined topic, including the effectiveness of VR intervention on a certain type of skill, such as attention detection, social functioning, or abstract concept and imagination. In contrast, narrative reviews on the use of VR in ASD research and practice account for only a tiny proportion. It is widely acknowledged that systematic reviews and narrative reviews provide complementary contributions [12,13]. They are both useful and indispensable in summarizing current knowledge and promoting further development in a newly emerging field. However, the fact that a substantial majority of existing review articles on VR and ASD adopt the systematic approach might indicate the existence of a spurious hierarchy of placing systematic reviews above narrative reviews on this topic, as has been pointed out in some research areas [12]. While the overwhelming emphasis on narrowly focused questions serves the purpose of solidifying positive evidence, its lack of broad perspectives may not be helpful in establishing a thorough and comprehensive understanding of using VR for ASD research and training. In this regard, we chose to present, in a narrative way, several selected topics on VR uses for ASD populations to reflect upon the current understanding of this burgeoning field. Our purpose is to adopt a broader perspective to showcase and discuss VR as an educational and interventional platform for individuals with and without ASD. Our target audience includes researchers and practitioners as well as families and the general public, who may have a shared interest in translational VR technology for autism treatment. Researchers who are concerned about the effect size of VR as an interventional tool are encouraged to read the systematic reviews in Table 1.

## 3. VR as a Powerful Tool in Education and Intervention

Though scientific literature has discussed at length the application and efficacy of VR [21,24,25], it remains a mysterious novelty due to relative inaccessibility. People may not be unfamiliar with its name, but often have only a vague idea of what VR means, and how it can serve pedagogical and interventional needs. This section provides background information, including theories and models that support its applications in education and rehabilitation.

### 3.1. Definition of VR

Different studies and disciplines have approached the definition of VR differently [19]. Augmented reality (AR) and mixed reality (MR) are often used interchangeably with VR despite distinctions. Specifically, AR adds virtual capability to the real-world environment, MR incorporates interaction between real and virtual elements, and VR offers the highest level of immersion, by replacing the physical world with an entire virtual world [29]. Their commonality lies in the use of virtual data to alter the physical world around the user. Considering that this cutting-edge technology is still under development and that many research studies in this field provide preliminary findings, we chose to be relatively flexible and inclusive in the definition of VR, covering research and practices that might involve a limited level of immersion, such as those using AR and MR.

### 3.2. Theoretical Underpinnings and Models for VR Training and Intervention

Theoretical support for the use of interactive media like VR for intervention stems from two basic facts. First, it simulates settings in the real world. Second, it provides enhanced experiences that are fully controlled and may be better suited for repeated practice. For instance, cognitive rehabilitation theory (CRT) is based on neuropsychological and cognitive psychological models [30]. As an integrated approach, involving individualized training of real-world tasks through a variety of techniques for daily functioning improvement [31], CRT emphasizes the significance of the individual’s social system and personal and environmental context [32], and acknowledges the complicated interplay of these contexts and rehabilitation techniques [33]. Following these fundamental ideas, researchers and practitioners have attempted to develop therapies and build models for clinical rehabilitation and intervention. For example, Dhamodharan, et al. proposed an evidence-based cognitive and occupational therapy using an interactive VR environment [8]. Their model analyzes three levels of decreasing functioning and cognitive impairment in children with autism, including attention, reasoning, emotion, social behavior, language understanding, and decision making, aiming to improve their mental states and life skills.

Pedagogical theories support VR being used as an educational tool in school activities. Classic pedagogical theories, including constructivist learning, situated learning, and engagement theory, all endorse the idea of VR integration into education [34,35,36]. The constructivist theory considers knowledge to be constructed through learners’ interaction with the environment. Situated learning holds that learning is realized through continuous participation in authentic activities in the community of practice. Similarly, the fundamental idea underlying engagement theory highlights the necessity of learners being meaningfully engaged in learning activities via interpersonal interaction and task completion. In these theories, VR integration is envisioned as the platform that enables full participation and facilitates all aspects of interaction and engagement. Furthermore, newly-advanced theories of pedagogy, such as experiential learning and affective learning, provide even greater support for the investigation of how VR technologies can be harnessed in education and learning.

The basic idea of experiential learning is to provide a learning environment or context for learners to actively experiment. It tests the hypotheses conceptualized from their previous experiences, and generates new knowledge and experiences for new situations. It is represented as an iterative learning cycle consisting of four steps, i.e., concrete experience (learners’ active experiencing and thinking in a given situation), reflective observation (analysis of observed outcomes of the experiencing), abstract conceptualization (situation understanding and hypothesis proposing), and active experimentation (hypothesis testing through active experimenting in new situations) [37]. Experiential learning has become one of the most widely applied pedagogical theories for VR integration into education, because interactive media technologies, especially immersive virtual environments (VEs), can allow learners to actively experiment and reflectively observe in a safe and authentic environment [38]. VEs can be better suited to, and advantageous for, promoting learning, as VR technology allows flexibility and control to remove competing and oftentimes conflicting sources of information from the real-world social and environmental context and to manipulate variables, such as break intervals and other motivational factors/incentives to solidify learning [39]. The realism of the VEs as a design feature of the technology can also facilitate the transfer of important skills into everyday lives [40,41,42].

Another important concept involves socio-affective mediation in the creation of VR learning environments. Affective learning theories have provided solid theoretical support for VR application in learning and education, as empirical evidence has demonstrated that VR positively influences the affective experience that learners perceive, enhances their learning engagement, as well as their motivation to learn, and ultimately leads to better learning effectiveness [43]. For instance, the Cognitive-Affective-Social Theory of Learning in digital Environments (CASTLE) proposes that social cues in digital materials and environment help activate social schemata in learners while reaping the benefits of enhanced social, motivational, emotional, and metacognitive processes [44]. Researchers, e.g., [44], supporting affective learning, purportedly introduce the impact of affective factors in the learning process. Kort, et al. [45] developed a model that took into consideration the interaction between emotions and learning and suggested that if a learner’s affective state is recognized and responded to appropriately, learning will proceed at an optimal pace. Similarly, Ip, et al. [46] proposed a pedagogical model for affective learning, namely, the Smart Ambience for Affective Learning (SAMAL) Model, which considers interplay between body, mind, and emotion during the learning process.

In recent years, interdisciplinary educators have started to work on practice-based theoretical models of VR in education. As target knowledge and skills are often multi-layered with different steps or levels of mastery, scalability or proper stratification, for programmatic progress to improve usability, is necessary. For example, Bambury [47] proposed the Depths of VR Model, which categorizes and differentiates between the different VR experiences available to students and educators. The model contains four progressive levels: Perception, Interaction, Immersion, and Presence. At the Perception level, which is the foundational level, learners are relatively passive and their experience needs to be supported and framed by well-considered pedagogy. From the Interaction level to the Immersion level, experiences become more engaging and student-led, and the potential for creativity and autonomy increases. Experiences at the Presence level are characterized by the sense of “being there” and have the potential to foster a “more visceral, emotive response” in students, which enables the deepest level of learning.

### 3.3. VR Answering the Needs of Both Neurotypical and Special Populations

VR is loaded with a diversity of capabilities, which can satisfy the different needs of normal people and special populations. For the neurotypicals, VR serves as a tool for professional training and school education. In training for some professions, such as pilots, VR-based environments simulating flying scenarios are used in place of real aircraft for lower operating costs and safety considerations. VR technology has also been developed to meet the requirements of pilot training, including short lag time, rapid update rates, and motion and force feedback [48]. In school education, VR technology offers various capabilities that contribute to better learning and education outcomes. It helps learners to visualize abstract concepts and their dynamic relationships and allows learners to visit and interact with people and events that are otherwise inaccessible or unfeasible because of time, distance, cost, or safety problems [34]. VR- and AR-based interactive learning environments have been found to be useful, especially for primary/elementary level education [49].

For special populations, VR can be developed into an idealized tool for intervention and rehabilitation by providing a real-life, but more “friendly”, environment. Individuals having special physical or mental conditions may have difficulty caring for themselves or controlling their behaviors and can thus feel awkward in real-life settings with other people. However, many of them have the need to improve their social interaction abilities for their day-to-day life in the real world. This dilemma can be approached through VR technology, which provides a safe and manipulable VE where intervention can take place in a customized and incremental manner under the control of therapists [40,50].

For children and adolescents with special needs, VR can offer substantial educational benefits in addition to its interventional advantages [24]. Education is a challenging issue for atypical children, since pedagogical design and frameworks for typical-developing (TD) children are usually unsuitable for them. During the learning process of children with learning disabilities or cognitive and perceptual impairments (including ASD), experiencing can play a more important role because they are often described as “concrete thinkers” who may find it difficult to comprehend abstract ideas or representations [51,52]. Affective factors may also have a larger influence on the learning results of children with special needs as they often suffer from emotion dysregulation and accompanied affective problems [53,54]. Therefore, their learning environments need to be experience-oriented, context-embedded, and should lay great emphasis on affective consequences and emotional responses. VR-driven pedagogical platforms offer the possibility of meeting these special demands. In VEs, children with special needs are exposed to diverse concrete experiences embedded in real-world contexts and can learn from mistakes without being discouraged or suffering real consequences. Moreover, VEs can enable them to experience and explore in ways that may be impossible for them in the real world, for example, allowing wheelchair users “to see how the world looks from a standing perspective” [52]. VR technology, with these user-centered features and capabilities, holds the promise to become a uniquely empowering technique in special education.

### 3.4. Showcases of Popular VR Products

VR has been developing rather rapidly, with cutting-edge technology quickly applied to commercial production, making VR products increasingly diverse and more affordable over time [17]. Currently, modern immersive VR environments are generally created by surround-screen projection-based displays, head-mounted displays (HMDs), or boom-mounted displays (BOOMs) [55,56].

Surround-screen projection-based displays, such as the Cave Automatic Virtual Environment (CAVE) [57], usually consist of several projection walls constructed to form an immersive environment, and motion trackers that track users’ head and body gestures. These installations allow a group of users to share virtual experiences in the same physical environment, which is particularly useful for education in classrooms where teachers tend to deliver VR learning materials to a class of students and keep them engaged in the immersive VE. However, due to the technical complexity of constructing such displays and the stringent requirements for the venue, it is quite difficult to apply them to large-scale general education [58].

VR headsets are among the most common VR devices in the market, ranging from the relatively inexpensive, but limited, Google Cardboard to more advanced HMDs, such as Oculus Rift, Oculus Quest, and HTC VIVE. Google Cardboard is a VR viewer that can be purchased at moderate cost and has been favored by school teachers because it is a stand-alone product that can be easily assembled and has a lower price [59]. However, its major disadvantage is that the VE created by Google Cardboard is actually a 360° image or video, where users cannot walk or move to approach surrounding objects. The virtual experience provided by later HMDs is more immersive and real-world-like. Equipped with advanced input devices (e.g., joysticks, gloves, trackers), users wearing HMDs can have a high degree of autonomy in the VE where they can move around and perform various actions in a pretty similar way to the real environment. In addition to the high level of immersion and autonomy, HMDs also have the advantage of being portable and space-saving, which makes them a superior option for large-scale education and training.

Another type of VR display is BOOM, a variation on HMD that is suspended from an articulated arm measuring the head position and is held to the user’s face with handles [55]. Compared with HMDs, users do not need to support the display on their head and BOOMs provide higher-quality images with a shorter lag time and are not susceptible to the influence of magnetic fields, thus enabling fast and accurate tracking [60]. BOOMs are also very convenient for users to switch between the virtual world and the real world. After a user releases the display, another user can observe the same images from the same perspective. Thus, BOOMs might provide some benefits for rigorously-designed experiments that require participants to be exposed to exactly the same stimuli. However, they have the disadvantage of restricted operating range, due to the space taken up by the supporting arms, and are comparatively less widely used for education and clinical practice.

As HMDs provide adequately immersive virtual experience and are convenient to purchase and install, they are considered to be the optimal option for large-scale education and intervention [56] and have indeed been utilized in a considerable number of empirical intervention studies on people with mental health disorders (including autism) [61,62,63,64]. Some researchers pointed out that when wearing an HMD, the user’s vision is totally isolated from the real surrounding world. This may raise concerns about the use of HMDs in the ASD population [65]. Nevertheless, recent studies have obtained encouraging results that individuals with ASD adapt well to wearing HMDs and are able to comprehend, learn, and interact in VEs [66,67,68].

## 4. VR Technologies and ASD

It is important to recognize that VR materials and contents used for the ASD population for educational and interventional purposes should not only be adapted to suit the immersive environment but also be combined with appropriate pedagogical design targeting the core symptoms of ASD [56]. Applications of VR technologies can yield significant benefits provided that they are grounded in the key characteristics of the ASD population. In addressing the core impairments of ASD, and improving the current condition of autistic individuals, the unique advantages of VR for ASD therapy, and the potential of VR for investigating social interaction, could play a crucial role.

### 4.1. Core Impairments of ASD and Its Increasing Prevalence

An impairment is considered to be core if it distinguishes the ASD population from TD individuals and those with other developmental delays [69]. Abundant research on ASD has identified core impairments in a variety of social aspects, including social communication and interaction, and speech and language [70]. Social communication skills cover a broad range of verbal and nonverbal abilities used in real-life and dynamic social interaction, such as emotion recognition, emotion regulation, and eye-to-eye gaze. The ASD population has difficulty understanding others’ emotions through visual and/or auditory cues [71,72], fails to regulate emotions appropriately and effectively [53], engages less in direct eye-to-eye contact [73], and shows atypical viewing patterns in social contexts [74]. Speech and language skills, which are crucial for successful social interaction, are also frequently reported to be impaired in individuals with ASD. They demonstrate prosodic deficits, such as aberrant use of pitch and stress patterns [75,76], and are particularly weak in the pragmatic use of language for communication, such as poor discourse organization and maintenance [77], difficulty in understanding the speaker-listener relationship [78], and inability to conform to conversational rules [79]. It is hypothesized that these pragmatic deficits, together with emotion processing problems, are related to fundamental impairments in the domain of theory of mind [70,80], i.e., the cognitive ability to explain and predict human behaviors in terms of mental states, such as desire, belief, and intention [81,82]. These core impairments in communication and language are found to be universal in children with ASD across ages and ability levels [69,83].

Recent years have witnessed an increasing trend in the prevalence of autism in the US, China, and other countries. Recent data show that one in 59 eight-year-old children in the US is diagnosed with ASD [84]. The newest result revealed a similar prevalence in China, at around 1% [85], and in South Korea, 2.64% (95% CI = 1.91–3.37) [86]. The prevalence in various countries and regions has shown an increasing trend over time in all age groups [87,88,89], which highlights a growing need for resources to provide care for the ASD population. The expenditure on a child with ASD, covering therapies, medical care, and special education programs, is estimated to be approximately $17,000/year more than for a child without ASD [90], imposing a heavy economic burden on the family. The current intervention approaches, although fruitful in improving ASD individuals’ life skills, are not always available for families, due to the high costs, and less expensive intervention paths are required to be developed to benefit families with diverse financial capacities.

### 4.2. Advantages of Incorporating VR in ASD Research and Therapy

Assistive and augmented technology, such as VR, with the aforementioned theoretical backing and various commercial products, can offer an effective and inexpensive means of practicing social skills and daily functioning both within and outside of therapy. Given the characteristics of ASD, these capabilities are particularly valuable for individuals on the autism spectrum.

Treatment on VR platforms is less stressful and would be less likely to increase anxiety or stress, problematic management of which is common in children and adolescents with ASD [91]. The social deficits associated with ASD can engender feelings of anxiety, especially in higher functioning ASD youths, who are aware of their social disability, and this overwhelming anxiety might, in turn, aggregate their social impairments [92]. For example, social anxiety resulting from unsuccessful attempts at communication and interaction may contribute to ASD individuals’ avoidance of social situations and lead to further isolation from their peers [93]. This negative impact of anxiety that tends to accompany real-world social skill practice, and may reduce the effect of social communication intervention, can be minimized through the employment of VR technology, which allows children with ASD to practice their social skills in real-life contexts without fear of mistakes or rejection that they commonly encounter in real-world face-to-face exchanges [94].

VR technology can be combined with gamified approaches to increase the motivation, attention, and focus of participants with ASD. Attention-deficit/hyperactivity disorder (ADHD), characterized by symptoms of inattention and overactivity, is one of the most common comorbid disorders in people with ASD [95]. Different from TD individuals who attend preferentially to social stimuli, such as people, faces, and body movements [96], individuals with ASD show an overall reduced social attention, which becomes more severe when the stimuli have a higher social content [97]. The inattention problem often hinders the process of research or therapy that participants with ASD take part in, leaving the work of researchers and clinicians floundering. This could be resolved through the addition of a gamified VR element to current practice, since the novelty of VR, together with the playability of gamified design, could arouse among many children, as well as adults with ASD, a stronger interest in the tasks they are going to accomplish, increase their investment in the training, and improve generalization [98].

VR gamified design can promote our understanding of the lower-functioning ASD population and the support that they need. A majority of existing studies and training programs have shown promising results. However, they tend to focus on ASD individuals with average or above-average IQ, and exclude those with low-functioning ASD [99]. So further efforts are required to explore and support the ASD population at the lower end of the IQ distribution, which demands more novel and elaborate approaches in experimental and interventional design [72]. Research on lower-functioning individuals can be tricky because the tasks that are manageable for TD participants, or higher-functioning ASD participants, might imply a higher level of difficulty and demand for lower-functioning ones due to their reduced cognitive abilities, which might result in a low task completion rate. This poses great challenges for researchers and clinicians as they are unable to test whether the ability required by the specific task is intact in lower-functioning ASDs and would then hesitate to determine whether such difficulties stem from autism or mental retardation or both [100]. This situation can be ameliorated by the adoption of VR technologies, combined with a gamified design that promotes task completion by offering concrete, fascinating, and enjoyable dynamic stimuli. Previous attempts in using VR games to teach emotions, and in VR music education, have reported the ability of children with low-functioning autism to complete VR game-like intervention [99] and recorded pretty high improvement for them [101], demonstrating that VR platforms can be especially beneficial for low-functioning autism children.

It has been well-documented that VR technology can offer considerable educational benefits for children and adolescents with special needs. A substantial proportion of people suffering from ASD are in childhood or adolescence. According to the fifth edition of the DSM criteria, the age of ASD onset is “early childhood” [1], and the symptoms usually persist through the school ages and are maintained into young adulthood [102], which renders the education of young people with ASD a primary concern and an intractable undertaking. Given the nature and severity of their disability, learners with ASD require carefully-designed individualized planning to obtain educational success, which brings considerable challenges in including students with ASD in general education classrooms [103], and calls for improvement and perfection of the special education system. The educational advantages brought by VR technologies could, therefore, contribute greatly to long-term support for the ASD population and would be illuminating for educators and policymakers working on special education services for children with ASD.

Apart from the above benefits for ASD, VR technologies also show superiority as a tool for research and treatment. For research, VR-based experiments have enhanced ecological validity, which is increasingly valued in the assessment of neuropsychological research, especially in the field of ASD [104]. Ecological validity, defined as the degree to which task performance corresponds to real-world performance [105] or the degree to which task performance predicts problems in real-life settings [106], has been viewed, to some extent, to conflict with the maintaining of experimental control [107]. Researchers supporting naturalistic approaches hold that many psychological assessments that use simple and static stimuli are ecologically invalid and are unable to generalize beyond restricted laboratory settings [108], while those emphasizing precise laboratory-based control argue that the ecological research approach lacks experimental control and the internal validity that are needed for scientific progress [109]. The tension between experimental control and ecological validity could be alleviated through the integration of VR, which allows precise presentation and control of dynamic perceptual stimuli in ecologically valid scenarios, thus increasing the generalizability of the findings whilst maintaining the same level of control as laboratory-based experiments.

Intervention programs employing VR techniques allow repeated practice and exposure, which is a key element in treatment [61]. In interventions such as social communication training, it is rare that participants successfully acquire the social interaction skill after practicing it only once. Compared with other therapeutic tools that might instruct participants to learn and respond in a rote manner, VR interventions provide the opportunity for repeated practice in dynamic social exchanges [110] and help participants learn by experiencing instead of memorizing. Additionally, on VR platforms, tasks and stimuli can be presented repeatedly and consistently without fatigue [111], avoiding the problem that usually accompanies task repetition by human tutors/instructors [112].

### 4.3. Potentials of VR for Investigating Social Interaction

Among possible applications, the one that receives particular attention from ASD researchers and therapists is the VR-based investigation of social communication and interaction, where ASD individuals tend to be especially impaired. VR offers great potential for social communication research and intervention as it can provide customized authentic scenarios and interlocutors, which are essential in real-life social communication, as well as the sense of being present at the scene of communication.

The social scenarios and contexts replicated in VEs can be carefully designed and controlled at the will of researchers and therapists to create whatever type of environment they want. For various research purposes, features of the real world can either be omitted, enhanced, or diminished, social relationships can be emphasized or modified, and the qualities and quantities of surrounding objects can be highlighted or weakened, increased or reduced [52]. The possible social scenarios that can be created through VR are arguably unlimited [113], including social introductions, initiating conversation, meeting strangers/friends, negotiating with a salesman, job interviews, working with co-workers, and managing conflict [61].

Virtual humanoid representations of people involved in the social scenarios, referred to as “avatars”, can be designed to serve as communicators, which carry out social communication with the user and give him or her hints on the communicational rules, and, as facilitators, which offer the user positive reinforcement upon his or her successful attempt to communicate or encourage further practice when the user makes mistakes [114]. The avatars can also be manipulated according to the requirements of researchers and clinicians; for example, the clinician’s voice can be morphed by software to sound like a young boy in order to match the avatar’s demographics [61]. Moreover, with the aid of artificial intelligence (AI), completely virtual characters can be created without the need for a therapist.

The sense of being present, which is positively correlated with the level of immersion [115], also contributes greatly to the potential of using VR for the investigation of social communication and interaction. Practical application is indispensable for the training of social communication skills, with the benefits of intervention, which are usually reduced when exposure to real-world social interactions is absent or inadequate [116]. Individuals with autism have reported a desire for more real-world practice after learning social communication skills in clinical intervention because they sometimes find it difficult to understand a social phenomenon until they see or experience it in a real-world setting [116]. Practicing a learned skill in a real-world context, and being successful in that situation, would also build confidence in people with ASD and help reduce their social anxiety [116]. Modern VR technologies and products, including surround-screen projection-based displays, like the CAVE, and HMDs, like Oculus Rift, with multisensory (e.g., visual, auditory, and tactile) congruent cues in the VEs, further enhance the sense of presence [115], and enable a fairly high level of immersion, rendering them viable tools for study and training of social communication skills.

## 5. Recent VR Applications in ASD Assessment and Intervention

VR technology gains theoretical support from rehabilitation and pedagogical theories with powerful and diverse capabilities. When it is instantiated in accessible and affordable commercial products, VR is a great alternative technique for ASD research and therapy and a promising methodological tool to investigate and improve social communication and interaction skills. The promising prospect of technology-driven pedagogical and intervention platforms has prompted researchers to integrate VR technologies into ASD assessment and intervention, with success, especially in terms of social communication deficits. This section illustrates recent evidence-based applications of VR technologies to ASD research and treatment, with a primary focus on the social aspects, including social functioning, emotion recognition, and speech and language. The key information of these studies is outlined in Table 2.

### 5.1. Social Functioning

Problems in social functioning (e.g., unemployment, impaired social skills, low social motivation, high social anxiety, social avoidance), are common in the ASD population [117,118]. There has been some clinical evidence that VR-based systems can have an increasingly positive impact on the social functioning of individuals with ASD [119]. With the aid of assistive technology, especially VR, participants with ASD showed improvements in social task performance [120,121,122], communication ability [123], sensitivity to social contingencies (i.e., the other’s responsiveness to one’s own behavior) [124], social competences and executive functions [125]. There is also evidence that in real-time, computer-mediated social space, people with ASD could perform social tasks equally well as controls, indicating that the use of VR interfaces could help compensate for their social disabilities [124].

In recent years, investigators have been developing VR agents, systems, and platforms to offer social functioning training to people with autism. For example, Bernardini, et al. [126] created an autonomous planning-based agent called Andy that inhabits a VE designed for real-world use at home and in schools. This agent is the main component of the Intelligent Engine of ECHOES, a serious game built to help young children with ASD acquire social communication skills [127]. In the game-like intervention program, Andy the agent, is always positive, motivating, and supportive and plays a diversity of roles, including a tutor who delivers visual and organizational support for ASD children, and a peer who provides them with customized interpersonal support and exposes them to positive interactions. The system, together with the agent, has been deployed in five schools in the UK and was proved to be effective in improving social behaviors in an eight-week intervention study involving 19 children with autism [126]. Autonomous virtual agents such as Andy could contribute to the intensive one-on-one support needed by ASD children, easing the demand for such support from clinicians and parents [126].

Considerable efforts have been made to alleviate the employment problem of individuals with ASD through VR-driven intervention systems. Burke, et al. [128] created a Virtual Interactive Training Agent (ViTA) system to offer practice in job interview skills required in interview conditions at various difficulty levels. The system includes six virtual human agents of varying ages and ethnic backgrounds, who can exhibit soft-touch, neutral, or hostile dispositions when asking 10 to 12 interview questions, and seven situational contexts, such as business office, hotel lobby, and warehouse breakroom, according to participants’ specific employment interests. In a preliminary study, the ViTA system has enhanced job interviewing skills in 32 participants with autism and developmental disabilities, with their face-to-face interview outcomes improved significantly after training, demonstrating its effectiveness as a tool for preparing young adults with autism for employment interviews [128].

**Table 2:** Empirical studies reviewed on VR applications in ASD assessment and intervention.

Authors, Year, Country of Study Origin	Study Type	Sample Size	Age (SD; Range)	Methodology	VR Equipment/ Platform Used	Main Findings
Social Functioning						
Bernardini, et al. [126], 2013, UK	Case study	19	N/M	Playing with the virtual agent for ten to twenty minutes, several times a week over an eight-week period	The IE of ECHOES	Some children have benefited from their exposure to the virtual agent and the ECHOES environment as a whole; there was an increase in almost all the social behaviors.
Burke, et al. [128], 2018, USA	Case study	22	23 (3.12; 19–31)	Training job interview skills using an interactive VR job interview practice system over a 14-week period	ViTA	The ViTA system improved face-to-face interview outcomes and is a promising intervention for preparing young adults with ASD for employment interviews.
Didehbani, et al. [110], 2016, USA	Case study	30	11.4 (2.7; 7–16)	Ten one-hour sessions of social skills training through interactive VR-learning scenarios	Second Life^TM^	Improvements were observed in emotion recognition, social attribution, and executive function.
Kandalaft, et al. [61], 2013, USA	Feasibility study	8	21.2 (2.7; 18–26)	Training social and social cognitive skills through VR scenarios for ten sessions over a five-week period	Second Life^TM^	There were significant increases after training in social cognitive measures of theory of mind and emotion recognition, and in real-life social and occupational functioning.
Russo-Ponsaran, et al. [129], 2018, USA	Feasibility study (case-control)	21	11.19 (1.27; 8–12)	Playing the role of a customized avatar and engaging in several challenging social situations for approximately 40 min	VESIP^TM^	Children with and without ASD understood and interacted effectively with VESIP, which demonstrated adequate internal consistency reliability; children with ASD scored lower on SIP domains than TD peers.
Smith, et al. [130], 2014, USA	Feasibility study (randomized controlled trial)	16	24.9 (6.7; 18–31)	Computer simulation of job interviews in VR for five sessions (two hours per session)	VR-JIT	VR-JIT participants had greater improvement during live standardized job interview role-play performances.
Stichter, et al. [131], 2014, USA	Case study	11	12.6 (0.7; 11–14)	Social competence training in computer-generated 3D VE for 31 sessions over a four-month period	iSocial	Social competence training was implemented in the iSocial environment at a high level of fidelity.
Strickland, et al. [132], 2013, USA	Efficacy study (randomized controlled trial)	11	18.21 (1.03; 16–19)	Interviews with virtual characters in JobTIPS computer program	JobTIPS	Youth who completed the JobTIPS employment program demonstrated significantly more effective verbal content skills than those who did not.
Zhang, et al. [133], 2018, USA	Feasibility study (case-control)	7	13.71(2.70; 7–17)	Playing games in a CVE for a single session that lasted approximately 60 min	A CVE platform and a set of CVE-based collaborative games	Children with ASD demonstrated improved game performance and trends in communication in a CVE.
Zhang, et al. [134], 2020, USA	Feasibility study (case-control)	20	13.39 (2.07; N/M)	Playing games in a CVE with their TD partner child and the intelligent agent respectively	CRETA, a CVE and intelligent agent	A moderate to high agreement was found in displayed communication and collaboration skills between human-human and human-agent interactions.
Zhao, et al. [135], 2018, USA	Feasibility study (case-control)	6 + 6 (two studies)	12.38(2.60; N/M); 12.12(3.59; N/M)	Playing collaborative games in Hand-in-Hand	Hand-in-Hand, a communication-enhancement CVE system	The system was well accepted by both children with and without ASD, and improved their cooperation in game play, and demonstrated the potential for fostering their communication and collaboration skills.
Emotion Recognition						
Bekele, et al. [136], 2016, USA	Usability study (randomized controlled trial)	6	15.77 (1.87; 13–17)	Emotion recognition in a social context with eye gaze, EEG signals, and peripheral physiological signals recorded in real-time	MASI-VR system	The System was useful in training core deficit areas for eventual better social functioning.
Frolli, et al. [137], 2022, Italy	Efficacy study (randomized controlled trial)	30	9.3 (0.63; 9–10)	Recognizing emotions and situations in a three-month VR emotional literacy intervention that involved the 3D projection of two sequences of scenes	A 3D viewer in VR	The group using VR showed shorter acquisition times for the use of primary and secondary emotions. VR can be a promising, dynamic, and effective practice for the support of basic and complex social skills of ASD individuals.
Ip, et al. [138], 2016, Hong Kong, China	Case study	52	N/M (N/M; 6–11)	Training in school-related social scenarios in four-sided CAVE for 28 one-hour sessions over a 14-week period	A 4-side fully immersive CAVE™ VR installation	Children showed significant improvements in emotion recognition, affective expression, and social reciprocity after training.
Kandalaft, et al. [61], 2013, USA	Feasibility study	8	21.2 (2.7; 18–26)	Training social and social cognitive skills through VR scenarios for ten sessions over a five-week period	Second Life^TM^	There were significant increases after training in social cognitive measures of theory of mind and emotion recognition, and in real-life social and occupational functioning.
Ke and Im [139], 2013, USA	Case study	4	9.75 (0.50; 9–10)	Completing social interaction tasks in a VR-based learning environment	A Second-Life-based social interaction program	There was an improvement in the performance of social tasks after VR intervention.
Kim, et al. [140], 2015, USA	Case-control study	19	11.1 (2.5; 8–16)	Recognizing basic emotions in a simulated real-world encounter with an avatar	V-REST	Children with ASD displayed significantly less approach behavior to positive expressions of happiness than TD children.
Lahiri, et al. [73], 2011, USA	Usability study	6	15.60 (1.27; 13–17)	Watching virtual classmates narrating personal stories and answering questions about the presentations	VIGART	There was an improvement in behavioral viewing and changes in relevant eye physiological indexes of participants while interacting with VIGART.
Lorenzo, et al. [141], 2016, Spain	Case-control study	20	N/M (N/M; 7–12)	Learning emotional scripts in VR scenarios	IVRS	Emotional behaviors improved in real school during the study because of IVRS.
Modugumudi, et al. [142], 2013, India	Efficacy study	10	11.6 (N/M; 7–19)	Six-month training in a CVE where emotions were displayed and participants communicated with the remote observer by expressing and recognizing the emotions	CVE	The CVEs were effective in training ASD children.
Yang, et al. [143], 2018, USA	Case study	17	22.50 (3.89; 18.06–31.08)	Training social and emotional skills in immersive role-play over 5 weeks for a total of 10 h	VR-SCT	The results provided evidence of the harnessable neuroplasticity in adults with ASD through an age-appropriate intervention in brain regions tightly linked to social abilities.
Speech and Language Training						
Bosseler and Massaro [144], 2003, USA	Efficacy study	8 + 6	N/M (N/M; 7–12)	Attending customized vocabulary lessons given by a computer-animated tutor	Baldi, a computer-animated tutor implemented in a Language Wizard/Player	Children with autism are capable of learning a new language within an automated program centered around a computer-animated agent and can transfer and use the language in a natural, untrained environment.
Chen, et al. [145], 2019, China	Efficacy study	11	4.81 (0.87; 3.33–6.90)	Learning to produce syllables through a three-session pronunciation training program	A computer-assisted 3-D virtual pronunciation tutor	The 3-D virtual imitation intervention system provided an effective approach to audiovisual pronunciation training for children with ASD.
Nubia, et al. [146], 2015, Colombia	Case study	6	N/M (N/M; 3–9)	Recognizing categories (e.g., animals, fruits)	An augmented reality mobile application	There was an increase in the appearance of verbal language after using the AR mobile application compared with the traditional method.
Saadatzi, et al. [147], 2018, USA	Efficacy study	3	7.33 (1.15; 6–8)	Learning sight words in an intelligent tutoring system	A desktop VE and a humanoid robot as the pedagogical agent	The intelligent tutoring system was effective in instructing sight words to children with ASD.

CVE = collaborative virtual environment; EEG = electro-encephalography; IE = Intelligent Engine; IVRS = Semi-Cave Immersive Virtual Reality System; MASI-VR = Multimodal Adaptive Social Interaction in VR; N/M = not mentioned; SIP = social information processing; V-REST = Virtual-reality Emotion Sensitivity Test; VESIP = Virtual Environment for Social Information Processing; VIGART = Virtual Interactive system with Gaze-sensitive Adaptive Response Technology; ViTA = Virtual Interactive Training Agents; VR-JIT = Virtual Reality Job Interview Training; VR-SCT = Virtual Reality Social Cognition Training.

Similarly, Smith, et al. [148] developed Virtual Reality Job Interview Training (VR-JIT), a program providing VR-simulated repetitive job interviews grounded on hierarchical learning, to help facilitate job interview skill training for people with neuropsychiatric disorders. Targeting eight job-interview performance domains, such as conveying oneself as a hard worker and sounding easy to work with, the system provides repeatable VR interviews for trainees to interact with a virtual human resources representative, offers them instant feedback to improve their responses, returns scores on key dimensions of performance, and allows review of interview responses. The feasibility and efficacy of VR-JIT have been assessed in a single-blinded randomized controlled trial involving 26 participants with autism, the results of which suggested that finding it easy to use and enjoyable, individuals with ASD showed improvements in interviewing skills after receiving training on the VR-JIT system [130] and achieved better vocational outcomes [149].

Another VR-enabled program targeting the employment issue was designed by Strickland, et al. [132], called the JobTIPS program, which offers five sections to guide trainees with ASD through the process of determining career interests, finding a job, getting a job, and keeping a job, and also provides other employment-related topics, like leaving a job. Results of a randomized controlled study on 22 participants with autism revealed that the JobTIPS employment program led to enhanced job interviewing skills and significantly more effective verbal content skills. Nevertheless, the author pointed out that their program was more effective in teaching “content” (i.e., producing *appropriate* verbal responses to the interview questions), rather than “delivery” skills (e.g., posture, eye contact).

Kandalaft, et al. [61] focused on more comprehensive aspects of social functioning and developed the Virtual Reality Social Cognition Training (VR-SCT) intervention, a semi-structured, manualized intervention that offers VR-based dynamic practice of meaningful social scenarios for young adults to improve social cognition, social functioning, and social skills. Scenarios in various social contexts were constructed to simulate common real-life social situations, including meeting new people, negotiating financial or social decisions, dealing with a roommate conflict, and interviewing for a job. A feasibility study, involving eight young adults with ASD [61], and another involving 30 ASD children and adolescents aged 7–16 years [110], were conducted to examine the effectiveness of VR-SCT and found that after 10 sessions of VR-SCT interventions, there were significant increases in ASD participants’ social and occupational functioning in real life, as well as a series of social cognition measures demonstrating positive impacts of the VR-enabled social skill training program on a wide range of social functioning skills and social abilities in individuals with ASD across ages. The treatment effectiveness has been further verified by recent investigation on the neural mechanisms of response to intervention in autistic participants receiving VR-SCT, which discovered that such interventions are not only useful in improving their social cognition and social skills, but also contribute to the strengthening of underlying brain networks that support their higher social functioning capacity [143].

In addition to the above VR social skill training systems, many other VR-driven platforms have also proved to be efficacious in enhancing social functioning and social understanding in ASD, such as Virtual Environment for Social Information Processing (VESIP) [129] and collaborative virtual environment (CVE)-based social interaction platforms, including iSocial [131] and CVE-based puzzle games [133,134,135].

### 5.2. Emotion Recognition

Difficulty in recognizing and understanding emotional expressions through multiple cues is one of the fundamental social impairments in the ASD population [150,151]. Children with ASD often show an atypical emotional development from TD children, manifested as a lack of empathy with other people and failure to react emotionally to other people’s states of mind [141]. Learning emotion recognition in VEs could remove such emotional barriers and obstacles for the ASD population, as VR training programs have been proved to be particularly helpful for them regarding emotion recognition improvement. For example, previous studies have reported enhanced behavioral performance [61], as well as neural predictors of change [143,152], in emotion recognition and theory of mind in participants with ASD after they completed the VR-SCT program. Similar results were obtained by Ke and Im [139], who observed a consistent and obvious increase in ASD children’s perceptions of emotion from facial expression and posture cues after a VR-based social interaction program, and Ip, et al. [138], who documented significant improvements in emotion recognition in over 100 school-aged ASD children after training completion in a CAVE-like immersive VR-enabled system. A recent study compared emotional training via the use of VR and traditional individualized training with a therapist and found an advantage for the use of VR, in terms of the acquisition time for the recognition of primary emotions [137].

In terms of emotion recognition, the level of immersion in the VE could influence the intervention effect. Lorenzo, et al. [141] conducted a randomized controlled study in which they designed an immersive virtual reality system (IVRS) to train and improve the emotional skills of 40 children with ASD. Their results revealed that, compared with the use of desktop VR applications, participants showed more appropriate emotional behaviors in the immersive VE and there was a significant improvement in their emotional competence after IVRS training. This finding is corroborated by the results obtained by Schwarze, et al. [153], who found that VR settings are motivational and useful for individuals with ASD to learn emotion recognition, especially under the condition that similar traditional approaches were transferred to a virtual context, e.g., virtual emotion cards. Furthermore, owing to the high immersion of VR-HMD technologies that provide an enclosing, three-dimensional, and 360-degree environment for emotion recognition learning, children with ASD were observed to start behaving in an “extroverted” way in their interaction and learning activities [153].

A few recent studies have attempted to integrate VR technology with dynamic psychophysiological signals to improve intervention approaches regarding emotion recognition. Lahiri, et al. [73] developed a VR-based dynamic eye-tracking system called Virtual Interactive system with Gaze-sensitive Adaptive Response Technology (VIGART) that can deliver individualized feedback according to the user’s dynamic gaze patterns during emotion recognition training. The system contained five social communication scenarios, where the avatars narrated their experiences on various topics, such as food, sports, travel, etc. Results of a usability study with six ASD adolescents confirmed that the VIGART can dynamically record eye physiological indexes, enabling objective measures of the user’s emotion recognition capability that could, in turn, guide the refinement of intervention strategies [73].

Modugumudi, et al. [142] conducted an electrophysiological study of two groups of autistic children (ten in each) receiving an intervention program with, and without, CVEs as assistive technology, to see whether children with autism could recognize basic emotions effectively in CVEs. The results showed that an emphasized early emotion expression positivity component at a latency of around 120 ms was identified for the CVE trained group, which clearly distinguished it from the trained-without-CVE group, indicating that children with autism had a significant improvement in emotion recognition owing to the CVE-based intervention.

Incorporating both eye physiological indexes and electrophysiological signals, Bekele, et al. [136] designed and developed the Multimodal Adaptive Social Interaction in VR (MASI-VR) system that facilitates individualization and adaptation for ASD emotion recognition intervention. The system presents controlled facial emotional expressions within conversational social contexts, tracking eye gaze as well as synchronously collecting electroencephalography (EEG) data associated with emotion recognition, while the users are receiving emotion recognition training and performing the emotion recognition tasks pre- and post-training. The viability and efficacy of this system have been verified through a randomized controlled study with 12 children with autism, supporting the idea of using task performance and eye gaze, and possibly other psychophysiological data, to enable real-time adaptation of ASD intervention in VR-based training systems [136].

Besides the above breakthroughs in terms of VR-enabled emotion recognition training, researchers have also sought assistance from VR technology to enhance the understanding of how individuals with ASD perceive and handle emotional expressions. For example, Kim, et al. [140] employed a novel measure called the VR emotion sensitivity test (V-REST) [154] to examine emotion perception and interpersonal distance in ASD with the aid of a joystick. While identifying the emotions expressed by virtual avatars, participants could position themselves close to or away from the avatars through the joystick. The study discovered that compared to TD children, children with ASD approached positive happy expressions significantly less, which suggests that they might display atypical social-approach motivation, or are less sensitive to the reward of positive socio-emotional events [140]. These results call for revision and updating of the social-motivation model of ASD [155,156].

### 5.3. Speech and Language Training

ASD populations often have delayed or impaired speech and language abilities, affecting both production and perception, which adds to their communication barriers [157]. Compared to the substantial efforts focused on VR-based training of social functioning and emotion recognition, less attention has been paid to applying this technology to speech and language therapy. The majority of the existing research and practice focuses on teaching discrete language components, such as vocabulary, grammar, semantics, and pronunciation, with the pedagogical and interventional platforms still limited to non-immersive VEs, such as desktop VE and AR.

A computer-based language-tutorial program that has been inspiring for later attempts in this area is the virtual talking head called Baldi, developed by Bosseler and Massaro to train vocabulary and grammar knowledge for ASD children [144]. Implemented in a Language Wizard, Baldi allows easy creation and presentation of language lessons concerning the identifying of pictures and the production of spoken words. An evaluation study was conducted to assess the effectiveness of this computer-animated tutor in vocabulary training and indicated that with the aid of the virtual agent, children with autism were able to learn language skills and could transfer the learned vocabulary to a new environment outside of the program [144].

Saadatzi, et al. [147] also developed a tutoring system targeting sight word instruction by combining VR technology and social robotics. This system featured a small-group classroom environment with a virtual agent as the teacher, and a humanoid robot as the peer, to facilitate observational learning, i.e., learning by watching others and imitating [158]. The effectiveness of the tutoring system was evaluated through an intervention study involving three participants with ASD who acquired, maintained, and generalized all the words that had been explicitly taught to them by the system and made fewer errors on the words that were also taught to their robot peer.

Nubia, et al. [146] resorted to AR to design and develop a mobile application that could serve as an alternative tool for semantic therapies to help improve semantic word knowledge in children with ASD. This application could stimulate oral and expressive language production by playing onomatopoeias or the sounds associated with animals and objects. Compared with conventional therapy, intervention using the AR mobile application led to an increase in the verbal language produced by the ASD participants, which was confirmed by speech therapists [146].

Apart from vocabulary, grammar, and semantics, the positive impacts of virtual elements and techniques on speech and language training in ASD have also been extended to the domain of pronunciation. Chen, et al. [145], incorporating the ideas of imitative learning [159], designed a 3-D virtual imitation intervention system to provide audiovisual pronunciation training for children with ASD. The computer-assisted 3-D virtual pronunciation tutor was able to present, in a multimodal and real-data-driven manner, the places and manners of Mandarin phoneme articulation, and has been proved to efficiently enhance the accuracy of Mandarin consonant and vowel pronunciation in low-functioning children with ASD [145].

## 6. Discussion

In this review, we aimed at presenting a broad perspective for researchers, practitioners, and the general public who are interested in incorporating VR technologies into educational and interventional platforms for children with ASD. We provided an overview of VR technologies and their benefits for ASD populations and reviewed recent evidence-based practices on VR uses in ASD that focused on social aspects. The application holds significant potential and considerable achievements have been recorded in terms of improving ASD individuals’ social communication abilities. However, there are technology- and design-related limitations that remain to be addressed, as well as controversial issues that must be taken into consideration in the design of future studies.

### 6.1. Limitations in Technology and Design

Though VR technology has improved tremendously in recent years, certain technical imperfections still exist, such as the graphic update rate, field of view (for HMDs), and lags between head tracking and visualization [160,161]. Besides these limitations, a particular technical challenge for VR applications is the simulation of behaviorally realistic virtual avatars. Current VR technology has been capable of creating vivid and lifelike avatars but they are inevitably different from real humans in either appearance or behavior. This would, to some extent, influence the responses of participants [25], in that if participants are constantly reminded that the people they encounter while using VR are not real, because the behavior of the avatars is not authentic enough, then the way they behave and respond in virtual worlds would diverge from that in the real world and could, thus, not be used as an indicator of their abilities. For example, in the study conducted by Parsons et al., one participant reported that he did not walk across the grass of a garden in the VE because that could make his shoes muddy; however, he walked between two people having a conversation in the cafeteria because they “weren’t real” and “it didn’t matter” [162]. Another technological limitation is the restricted range (usually a few square meters) within which participants have to use the interaction and tracking devices in order to maintain a good connection and interaction with the VE. These technical restrictions might limit the level of immersion provided by a VR system and result in a lower experience of presence [163].

In addition to the above technical limitations, inadequacies exist regarding learning content and activity design. Despite the promising results of current successful attempts in VR-based training in ASD, the VR learning content used in many existing studies focused on scenarios and situations that were too confined [164], and the skills being taught were often procedural and strongly rule-based, with inadequate emphasis laid on the skills required in relatively unpredictable social situations [9]. Another problem is that a certain number of training activities in current VR interventions are ill-devised, including only repeated and tedious practice that is likely to bore ASD children after the initial novelty wears off. One possible solution to this issue is to add recreation value to the VR training systems by borrowing serious game design concepts [165] from the entertainment sector. More enjoyable content and increasingly engaging activities would also attract more participants to the training systems, which can prevent the drawback of inadequate statistical power caused by the small number of participants in most previous feasibility and validity studies [166], helping researchers draw more convincing conclusions on the effectiveness of VR-based intervention systems.

### 6.2. Current Disputes

There have been disputes over the application of VR technology to ASD research and therapy. They cover mainly three aspects, i.e., challenges to the veridicality assumption, safety concerns, and ethical considerations. Some researchers have questioned the authenticity and veridicality of VEs that have provided a strong argument for the integration of VR-based design into various educational and health fields [25]. The potential and strengths of using VR to investigate social communication and interactions are grounded on the fundamental belief that VEs provide realistic and authentic experiences that mirror real-world behaviors and responses. However, this assumption may be open to question because the degree of perceived realism in VEs can be affected by many factors, including the features of VEs (e.g., the degree of autonomy allowed in the VEs, how real the avatars and the scenarios are [167], the way of interaction between users and systems [168], whether the avatars are controlled by humans or computers [169]), and the background characteristics of users (e.g., whether users have postural instability, whether they are susceptible to motion sickness [170]). The VR technologies adopted by many existing studies, such as AR and desktop VR, are actually non-immersive and provide rather limited autonomy. Although they have taken a step further, compared to the traditional simple and static stimuli, it is still debatable as to what extent the responses recorded in these VEs are generalizable to real-world responding and interpretation [25].

Moreover, even for the highly immersive VEs, created by HMDs or CAVE-like systems, which are among the most advanced VR techniques so far, technological limitations still exist regarding the level of influence of the perceived realism of VR experience and the transfer of learning to real-world contexts, which leaves plenty of room and possibilities for future improvement. For example, Parsons [25] suggested that there are at least two disparate directions for future efforts to move the field forward: to increase the realism of VEs and pursue extreme veridicality of VR technology, or to diverge from the pursuit of pure veridicality and consider the needs and preferences of the users more. Within the scope of ASD research and intervention, the latter perspective would lay more emphasis on questions, including the following: For people with ASD and their families, what elements in VEs are the most important and would be particularly useful to support and enhance their communication and learning? Do the degree of realism and the veridicality linearly correlate with the effectiveness of VR-based ASD training systems or is there a breakpoint at which the effectiveness plateaus? If the breakpoint exists, what degree of authenticity is optimal so that people with ASD and their families could benefit the most from VR technology without spending too much time and effort seeking cutting-edge equipment, or waiting for further technological development? There is a need to inquire into these aspects directly and in more detail in order to really understand how VR technology can positively influence the ASD population’s learning, well-being, and life quality.

Safety risks, both physically and psychologically, might be associated with the use of VR. A common physical safety concern associated with VR experience is cybersickness, which can cause fatigue, malaise, and dizziness, or even elicit a series of symptoms, such as eye strain, nausea, and bodily disorientation [171,172]. In addition to the possible physical discomfort, some researchers pointed out the potential psychological safety risks accompanying, but not unique to, the use of VR, suggesting that, similar to the problems documented in people engaging in excessive use of video games, extended and continuous use of VR might be linked with certain mental uneasiness [173]. These safety risks have led to some disputes over the use of VR in the ASD population, but rather than discouraging its application, we could instead carefully consider and control these risks in the design of future studies. For example, to minimize the consequences of cybersickness (including symptoms of nausea, vomiting, drowsiness, headache, loss of balance, and problematic eye-hand coordination), risk assessment based on predictive questionnaires, such as the Motion Sickness Susceptibility Questionnaire (MSSQ-Short) [174], ought to be conducted prior to training or experiments, and immediate reports of discomfort, as well as timely support from therapists, are necessary during the initial use of VR. For ASD trainees who have no previous exposure to VR, transition periods could be arranged to help them gradually adapt to VEs. As for the concern about psychological safety, the duration of exposure to VR should be strictly controlled in both research and therapy sessions to prevent the negative impact of excessive, and inappropriate, use from overshadowing the benefits of this technology.

Apart from the challenges to veridicality and the safety concerns associated with VR, ethical considerations also provoke extensive discussion, among which the most debated one might be the question of privacy and confidentiality during data collection [173]. In the process of VR-enabled research and intervention, eye-movement patterns, facial expressions, and body responses and reflexes, which constitute one’s distinct “kinematic fingerprint” [172], would be recorded. Given the commercial nature of many VR products, this personal information of participants could be accessed by companies that deliver the VR software, which tend to state in their privacy policy that user data may be collected. Therefore, researchers and therapists using VR technologies need to provide participants with clearly-stated informed consent forms to ensure that they are aware of, and consent to, the data collection before participation.

### 6.3. Future Directions

Since the first employment of VR technologies on the ASD population in the 1990s [65], there has been a significant increase over the past decades in the number of studies making similar attempts [18]. The literature has seen increasing recognition of the benefits and potentials of VR to facilitate learning, especially in social environments for individuals with autism. However, there is still much work that remains to be done in this research area. Future efforts can be directed towards application expansion and improvement, technology enhancement, and brain-based research and theoretical model development. The contributions of these basic and applied studies would be at least three-fold: benefiting the ASD population, reducing the workload of therapists, and facilitating the advancement of VR technology, as well as theoretical modeling of VR applications, with beneficial social and cultural consequences.

#### 6.3.1. Application Expansion and Improvement

Existing VR-based training systems and platforms have paid much attention to improving ASD individuals’ performance in social functioning, emotion processing, and speech and language. However, a majority of them treated these socially important aspects independently and designed training programs that centered around one specific aspect; few have taken an integrated view of these skills to systematically enhance social communication in ASD. Within each aspect, the research was also discrete and divided. For example, VR-based intervention programs and systems for speech and language training have focused on grammar and vocabulary, semantics, or pronunciation, but rarely attempt to integrate these components of language communication skills into one training system. Many training programs, though proven to be effective in improving ASD trainees’ specific skills, often meet with limited success in enhancing the overall social performance of the ASD population [175,176,177]. One probable explanation for this problem is that intervention and training that involves correlates of different target skill domains is often missing from the research and treatment efforts in ASD, and even though these correlates are included in some programs, they are not always trained systematically [176]. The “transactive” relationships between different behavioral domains [178] indicate that improvements in one domain contribute to progress in other domains and delay in any one could affect development in the others [179,180]. For example, VR training programs aiming to improve employment-related skills in ASD may lead to better use of strategies by participants with ASD during job seeking, but their actual overall performance might improve little, due to remaining weakness in emotion perception or speech production and understanding [132]. Effective intervention in social communication in ASD thus not only requires training in specific social aspects, including social functioning, emotion processing, and speech and language, but also calls for integrative efforts to resolve the difficulties that correlate these domains.

To systematically improve social communication performance that involves various social domains, future VR-based ASD intervention systems could lay more emphasis on the training and learning of prosody, which plays a vital role in a range of communicative functions (linguistic/grammatical, pragmatic, affective, etc.) [181] and the correct use of which, promoting peer interaction and socialization, is fundamental for both personal development and social integration of individuals with ASD [182]. Prosody functions at three major levels (not mutually exclusive) to enable smooth social communication: *grammatical* prosody is crucial for the expression of semantic meaning (e.g., resolving semantic ambiguity); *pragmatic* prosody conveys the speaker’s intentions (e.g., emphasizing certain information); *affective* prosody implies the speaker’s emotions or affective states (e.g., feeling happy or sad, relaxed or anxious) [183]. These functions cover a wide range of both linguistic and socio-affective domains, which means that atypical use and misunderstanding of prosody would greatly undermine efficient social interaction and language communication. More research and intervention studies are thus recommended to combine VR technology with integrated training in prosody, both linguistic and affective, and its combination with grammar, semantics, and pragmatics to improve socio-affective and linguistic skills, and ultimately enhance overall social communication in ASD. Considering the fact that emotions are conveyed in multiple channels and modalities (facial expression, gesture, affective prosody, and semantics), an integrated framework of language, emotion, and cognition needs to be developed to guide the work [184]. In this regard, embodied cognition theories can provide some foundational work with respect to potential issues and challenges in perception–action binding and perspective-taking in ASD [185].

Since a large proportion of ASD individuals are in childhood or of school age, ideas in the field of education would be helpful for alleviating ASD core symptoms over the course of development. Existing research on VR-based ASD training has benefited much from pedagogical theories [147] and future exploration could continue integrating philosophy in education and learning to develop better VR-based training programs for ASD. For example, Iovannone, et al. [186] proposed six essential components to be included in an effective educational program for ASD students: individualized support and services for students and families, specialized curriculum content, systematic instruction, comprehensible and/or structured environments, a functional approach to problem behaviors, and family involvement. These components, especially the first two, could be well-incorporated into VR-driven training programs by providing language- and cultural-specific individualized training packages to enable more effective targeted intervention.

Future design of VR training systems, especially for social communication training, needs to show more consideration for users’ language and cultural backgrounds. For example, it has been hypothesized that prosodic deficits in the ASD population reside primarily in the pragmatic and affective aspects, with grammatical aspects relatively spared [183]. However, this conclusion was mainly drawn from data collected from ASD individuals speaking English or other non-tonal languages, where syllable-level prosodic variations (or lexical tones) do not distinguish lexical meaning. For tonal language speakers with autism, atypical perception of lexical tones and vowel exaggeration has been observed [187,188], which indicates impairment in grammatical-prosodic processing in addition to their pragmatic difficulties [189]. Therefore, whether grammatical functions of prosody are impaired among the ASD population is disputed and is likely to differ, due to the influence of language backgrounds, so the design of future VR-based practice should take into consideration this language-specific problem and be tailored to ASD individuals according to their language backgrounds. Similarly, cultural factors should also be taken into account in future exploration in this area as many social behaviors are culture-dependent. For instance, training in the social skills of people with ASD usually encourages them to make eye contact with their communication partners. However, looking elders directly in the eye is regarded as disrespectful in some countries, such as India and Bangladesh [190]. It is thus crucial to design the training scenarios and activities with an eye to the cultural backgrounds of ASD users.

The heterogeneity within the autism spectrum implies that even if with the same language and cultural background, each individual with ASD is still unique and a specific intervention or treatment may not always be the best for the whole population [191]. The effectiveness of the intervention depends largely on how accurately it targets the specific vulnerabilities of the ASD participant, so it is necessary to offer individualized VR-based training and intervention that is able to cater to the preferences of each ASD individual. In conventional intervention, therapists tend to adjust the intervention paradigm according to the ASD individual’s specific cognitive and behavioral conditions [114]. Future development of VR-based training systems for ASD should also take into consideration the adaptation of skill training to the user’s specific needs. For example, research on emotion processing in ASD has produced very mixed results, with disagreement persisting over whether it is impaired in ASD individuals [71]. The heterogeneous performance across tasks and across individuals (even in one single study) suggests that emotion processing may not be universally impaired in ASD and the degree of deficits may also vary across individuals [192]. VR-based training systems could then incorporate AI assessment procedures to automatically examine the degree of certain deficits in the specific user and adjust the subsequent training plan according to the assessment results. For instance, for users with ASD who are assessed to have particular difficulty perceiving and understanding certain emotions, the VR training system may correspondingly adjust the proportion of training in emotion processing and provide targeted practice to promote optimum learning of relevant social skills.

For the same person, the condition may also change over time. In the short term, the performance during one intervention session would fluctuate and calls for dynamic and real-time adaptation. In the long term, the variation in the different stages of development also requires intervention objectives and strategies to be tuned accordingly. For example, school-aged autistic children may need more instruction in coping with bullying, whereas young adults with ASD would desire extra training in employment skills, such as finding and keeping a job. Besides the change in intervention-related needs, the cognitive level and the language ability of the ASD user might also improve over the course of development [193,194]. Then, in order for the intervention to be continuously effective, the learning content, the language material, and the activity design in the VR training systems should be updatable along with the developmental change of the ASD user.

#### 6.3.2. Technology Enhancement

As illustrated in the previous section, there is much room for future improvements to VR-driven training systems for ASD. Potential breakthroughs in all these directions require corresponding enhancement of VR technology to facilitate the application expansion. For example, researchers and engineers in VR technology development should investigate how to combine VR technologies with AI and machine-learning techniques to promote customized intervention and provide tailored support. Automatic recognition and assessment technologies, including automatic speech recognition and evaluation [195,196], and automatic assessment of cognitive and emotional states [197], need to be embedded into the VR training systems, to measure and track users’ abilities and states and to deliver immediate and timely feedback prior to, during, and after training. The incorporation of accurate, intelligent, and efficient automatic evaluation techniques can greatly ease the workload of speech-language and cognitive-behavioral therapists and, more importantly, offer much-needed assessment and intervention services for the ASD population living in less developed regions that lack trained and qualified therapists.

Future exploration may expand the functionality of VR so that it can be part of big data that promotes worldwide collaboration on autism research and facilitates data-driven discoveries. The ideal big data for autism research should be both “broad” and “deep”. Broad data is characteristic of large sample sizes, whereas deep data involves analysis of multiple levels of information collected on the same individuals, including behavior and development, neural systems, and outcomes of clinical treatment [198]. VR training platforms can be a perfect channel for gathering such “broad” and “deep” big data owing to their capability to collect dynamic and detailed behavioral and neural data at various stages of training and intervention. Future VR training systems can be improved to not only enable recording of multi-level data of ASD trainees, who have given informed consent, but also allow uploading of these data onto cloud storage space, as well as later downloading of the data by authorized users. Furthermore, researchers can draw from techniques and strategies that are commonly employed in designing, building, and managing a database and work out efficient ways of VR data pooling and collating to develop secure and searchable VR-based multi-level ASD data repositories for research purposes. Such databases can be very helpful in providing comparison and reference for ASD assessment and diagnosis, as well as furthering our understanding of heterogeneity in autism [198].

Another direction of function expansion for future VR-based ASD training systems is to provide ASD individuals and therapists with personal data banks for monitoring progress and informing subsequent intervention decisions. The training systems can be enhanced to regularly create and push visualized personal reports that document the ASD user’s progress and status quo; for example, how much the ASD trainee has progressed in certain skills since the last time, what skills he or she has improved in the most, which aspects require further practice in later training. This kind of feedback would be encouraging for ASD individuals and their families by assuring them that conditions are indeed improving and that they should persevere with the training. A more detailed personal data bank would also be useful for therapists, as information on pretreatment characteristics is often needed to individualize an intervention plan [114].

#### 6.3.3. Brain-Based Research and Theoretical Model Development

Existing evaluation of the treatment effectiveness of VR-based training in ASD relies primarily on behaviors, such as improved performance in tasks or tests, with limited evidence for the neural changes underlying these behavioral gains. Although a few pioneering studies have attempted to examine how therapeutic responses to VR training reflect brain changes in ASD [143], it remains to be answered whether VR can support the development of brain networks of ASD individuals. Brain-based research is crucial for VR application to ASD because brain data is an indispensable level of “deep” big data [198] and plays a crucial role in the substantiation, revision, and updating of theoretical models in ASD research [199]. Future investigation is thus recommended to utilize neurophysiological and neuroimaging techniques to track and measure brain responses of ASD participants before, during, and after VR training to evaluate the efficacy and limitations of multi-modal VR-based training from the perspective of brain plasticity, and to elucidate the neural underpinnings of behavioral improvements, both at the group level and the individual level.

Data obtained from brain-based research is also useful for the theoretical modeling of VR technology and its applications. Existing theoretical models of VR technology in ASD mostly focus on multiple levels of cognitive and behavioral enhancement [8] and few of them have touched upon how the brain or neural mechanisms of ASD individuals would evolve due to exposure to VR training. Future efforts could extend the existing models by linking brain activities and changes with behavioral improvements at multiple levels or developing new models that expound the influence of VR training on brain changes in ASD individuals at different developmental stages. In particular, specific concepts and issues, such as the understanding of self vs. others, and the multi-channel and multimodal nature of social signal processing that can inform biological, psychological, and neurophysiological underpinnings of social communication, need to be clarified. Such social neuroscience models, incorporating both behavioral and neural aspects, will provide insightful guidance for the designing, developing, and evaluating of VR-based ASD training platforms that can be potentially added to traditional behavioral therapies and educational settings with great social and cultural consequences.

## 7. Conclusions

The present review provides a synopsis of the use of newly-emerging VR technologies as educational and interventional tools for the ASD population. Grounded in mainstream rehabilitation and education theories, platforms and equipment based on VR technology show advantages in training of social communication and interaction skills. Evidence-based practices demonstrate that incorporating VR into therapy or training programs is effective in improving the social aspects of performances among individuals with ASD. Participants have shown remarkable improvement in social functioning, emotion recognition, and speech and language after VR-based intervention. For future studies in this field, technology- and design-related limitations are yet to be addressed, and controversial issues, such as safety concerns and ethical considerations, need to be taken seriously. Research efforts should, thus, be directed to application expansion and improvement, technology enhancement, and brain-based research and theoretical model development. Moreover, the majority of VR systems and related research targets users of non-tonal languages, with little consideration of cross-cultural and cross-linguistic differences. As tonal languages are estimated to represent 60-70% of the world’s languages [200], more efforts and studies are needed to include autistic children speaking tonal languages, so that a more global picture, having linguistic and cultural diversity, can be obtained for VR-driven education and intervention.

## Figures and Tables

**Table 1:** A summary of existing reviews on the use of VR for ASD intervention.

Authors	Publication Year	Review Article Type	Journal/ Conference	Focal Topic	Participants Covered	Time Period of the Included/ Covered Studies	Proportion of Studies Covering Tonal Language Speakers with ASD
Banire, et al. [14]	2017	Systematic review	*2017 7th IEEE International Conference on Control System, Computing and Engineering (ICCSCE)*	Attention detection and measurement in VR based learning intervention	Children with ASD	January 2008 to May 2017	18.18%
Bellani, et al. [15]	2011	Narrative review	*Epidemiology and Psychiatric Sciences*	Benefits of VR in supporting the learning process in ASD, particularly related to social situations	Children and adolescents with ASD	1996 to 2010	12.50%
Berenguer, et al. [16]	2020	Systematic review	*International Journal of Environmental Research and Public Health*	Impact of AR through social, cognitive, and behavioral domains in children and adolescents with ASD	Children and adolescents with ASD	January 2010 to April 2020	20%
Bradley and Newbutt [17]	2018	Systematic review	*Journal of Enabling Technologies*	Use of VR-HMD for educational assessment, approaches and interventions in ASD	Children and adults with ASD	1996 to 2017	16.67%
Dechsling, et al. [11]	2021	Systematic review	*Research in Developmental Disabilities*	Applying Naturalistic Developmental Behavioral Interventions (NDBI)-approaches in VR	Children and young adults with ASD	2010 to April 2020	10%
Dechsling, et al. [18]	2021	Scoping review	*Journal of Autism and Developmental Disorders*	VR and AR technology in social skills interventions for individuals with ASD	Mostly children and adolescents with ASD	2010 to February 2021	20.41%
Glaser and Schmidt [19]	2021	Systematic review	*International Journal of Human–Computer Interaction*	Design characteristics of VR systems designed as intervention or training tools for individuals with ASD	Mostly children and adolescents with ASD	1995 to March 2020	7.41%
Herrera, et al. [20]	2006	Narrative review	*Technology and Disability*	Abstract concept and imagination teaching through VR in ASD	People with ASD	1996 to 2006	0
Karami, et al. [21]	2021	Systematic review with meta-analysis	*Frontiers in Psychiatry*	Effectiveness of VR on the rehabilitation and training of individuals with ASD	People with ASD	Until October 2019	15.15%
Lorenzo, et al. [22]	2019	Systematic review	*Education and Information Technologies*	Application of immersive VR for students with ASD	People with ASD	1990 to 2017	9.09%
Mak and Zhao [23]	2020	Systematic review	*Interactive Learning Environments*	Application of VR on skill-specific performance in ASD	People with ASD	January 2012 to February 2018	0
Mesa-Gresa, et al. [24]	2018	Systematic review	*Sensors*	Effectiveness of VR-based intervention in ASD	Children with ASD	January 2010 to February 2018	19.35%
Parsons [25]	2016	Narrative review	*Educational Research Review*	Veridicality of VR for autism research	People with ASD	Until August 2016	4.00%
Shoaib, et al. [26]	2017	Systematic review	*2017 17th International Conference on Computational Science and Its Applications (ICCSA)*	Role of information technology in improving behavioral, communication, and social skills in ASD	Children with ASD	Until April 2016	N/A (included studies not explicitly listed)
Thai and Nathan-Roberts [27]	2018	Systematic review	*Proceedings of the Human Factors and Ergonomics Society Annual Meeting*	The most important social skills that VR systems should aim to train and the most helpful measures that would best assess the changes made	People with ASD	Until October 2017	N/A (included studies not explicitly listed)
Wang and Reid [28]	2011	Narrative review	*Neuroepidemiology*	Current status and use of VR for children with specific neurodevelopmental disorders	Children with ASD, ADHD, or cerebral palsy	2000 to 2011	0

## Data Availability

Not applicable.
